# Consortium management structures, processes, and approaches: The DELTAS Africa example

**DOI:** 10.12688/wellcomeopenres.17721.1

**Published:** 2022-04-21

**Authors:** Nadia Tagoe, Sassy Molyneux, Justin Pulford, Sam Kinyanjui

**Affiliations:** 1 Kwame Nkrumah University of Science and Technology, Kumasi, Ghana; 2 KEMRI Wellcome Trust Research Programme, Kilifi, Kenya; 3 Nuffield Department of Medicine, University of Oxford, Oxford, UK; 4 Department of International Public Health, Liverpool School of Tropical Medicine, Liverpool, UK; 5 School of Business Studies, Strathmore University, Nairobi, Kenya; 6 Bioscence Research Centre, Pwani University, Kilifi, Kenya

**Keywords:** Consortium, Management, Structures, Processes, Approaches, Health, Research, Capacity Strengthening

## Abstract

**Background:** Global efforts to strengthen health research capacity in low- and middle-income countries (LMICs) have intensified in the past few decades, and these efforts are often implemented by consortia. Our review of the literature indicated that reports on health research capacity strengthening (HRCS) consortia have primarily focused on programme outputs and outcomes while management processes and their contributions to consortia goals have received little attention. This qualitative study sought to identify the consortium management processes employed by 10 DELTAS Africa consortia, factors influencing these processes, and leaders’ consortium management experiences.

**Methods:** We conducted 24 key informant interviews with the directors and programme managers of all the 10 DELTAS Africa consortia, and funding actors who worked closely with the consortia. The interviews were supplemented by reviews of DELTAS and consortium-specific documents. Data were analysed using the content analysis approach.

**Results: **The consortia studied employed similar management processes but adopted different strategies in executing these processes. Study results indicate that decision-making in consortia is not always a straightforward process as leaders were often faced with dilemmas when determining management strategies to adopt, and often tried to balance multiple factors which were not always aligned. This was demonstrated as consortia selected partners, determined goals and activities, assigned roles and responsibilities, allocated resources, established governance and partner management systems, and coordinated and monitored consortia activities. Factors that influenced the choice of processes and approaches included previous experiences, funders expectations, and the pressure to deliver research outputs. Consortia’s unique approaches to management were due to varying contexts and influences and indicate that management decisions are nuanced and cannot easily be formularized.

**Conclusion:** The study has highlighted the importance of flexibility in consortium management and the need to generate research capacity strengthening (RCS)-specific guidance that can assist consortia in resolving dilemmas and making appropriate management decisions.

## Disclaimer

Parts of this study were included in the PhD thesis of the leading author, available from the Open University on Open Research Online: https://doi.org/10.21954/ou.ro.00012985 ^1^.

## Introduction

Advancing research is a critical component of the global health agenda. This realisation has intensified health research capacity strengthening (HRCS) efforts, particularly in low- and middle-income countries (LMICs) ^2^. Capacity strengthening programmes are often implemented by consortia comprising individual and organisational partners with variable resources, expertise, and experience, working together to achieve a common goal ^3, 4^. The increasing investments in HRCS consortia have heightened the need for closer scrutiny as part of assessing their effectiveness ^5^.

Our review of the literature revealed that although there is a growing body of work reporting consortia activities, there is little published evidence on consortium management and its role in the achievement of outcomes ^6^. The literature on HRCS consortia in general has focused on programme activities and outputs ^7– 9^, and there tends to be an emphasis on performance indicators such as trained researchers, publications, and grant awards ^10– 13^. While important to assess outputs and outcomes, it is equally essential to assess the processes involved to determine how and why the various outputs and outcomes are realized ^14^. Consortium management involves a series of steps and actions taken to establish and run core consortium activities including partner selection, resource allocation, and activity monitoring to deliver the specified outputs and outcomes. These complex management processes of coordinating partners, activities, and institutional systems have potentially important implications for consortia outcomes.

This paper describes the management structures and processes used by 10 consortia participating in the Developing Excellence in Leadership, Training and Science (DELTAS) Africa HRCS Initiative administered by the African Academy of Sciences (AAS) ( https://www.aasciences.africa/aesa/programmes/developing-excellence-leadership-training-and-science-africa-deltas-africa). We discuss how the consortia approached these processes and factors that influenced their management practices as a first step towards understanding the relationship between consortia processes, practices, and programme outcomes.

## Methods

### Ethical approval

Ethical approval for the study was obtained from the Kenya Medical Research Institute (KEMRI) Scientific and Ethics Review Unit with approval number KEMRI/SERU/CGMR-C/109/3591. Written informed consent was obtained from each study participant and the study was carried out in accordance with approved ethical guidelines. As a result of the unique characteristics of participating consortia and the small number and distinct roles of study participants, anonymization of study data does not adequately ensure the protection of participants. Hence, the request for permission from the AAS to engage the DELTAS consortia and the application for ethical approval included a statement on restriction of the raw data to the study team. This study was completed as part of a PhD project, and a full description of the methodology and larger project is publicly available ^1^.

### Study design and setting

We employed an exploratory qualitative study design set within phase one (2015–2021) of the DELTAS Africa Initiative which was administered by the African Academy of Sciences (AAS). This initiative was of interest because it involved Africa-led consortia, which represented new thinking regarding the leadership of HRCS efforts. This phase involved 11 African-led programmes aimed at strengthening health research capacity on the continent through four strategic areas: enhancing scientific quality, research training, scientific citizenship, and research management and environment ^15^. The programmes were hosted by 11 lead institutions comprising six universities and five research institutes based in both anglophone and francophone countries across sub-Saharan Africa: four in Eastern Africa, three in Southern Africa, and four in Western Africa ( https://www.aasciences.africa/aesa/programmes/developing-excellence-leadership-training-and-science-africa-deltas-africa#grantees). The programmes were driven by a theory of change which mapped out the expected outcomes and measurement indicators for the four strategic areas. The training of researchers (primarily Masters, PhD, and post-doc) was the main focus of this DELTAS phase. 10 of the programmes were implemented by consortia, and each consortium comprised an African lead institution and other African and international partner institutions. Consortia sizes ranged from four to 14 partner institutions, although each consortium worked with additional collaborators where required. All 10 consortia participated in the study.

### Data collection and analysis

Data were collected through key informant interviews and complemented with a review of relevant DELTAS and consortia documents. The document review preceded the interviews to help improve the focus and efficiency of the interviews. First, documents on the DELTAS Africa Initiative including the call for proposals, funder terms and conditions, submitted proposals, award letters, and annual reports were sourced from the AAS and reviewed using a checklist (Extended Data File 1) ^16^. Next, each consortium was given a template to provide relevant consortium data including composition, goals, governance and management structures and teams, functions, and activities (Extended Data File 2) ^16^. Extracted data were categorised according to the consortia and presented in an MS Excel (Version 1702) document.

We then conducted 24 key informant interviews with 10 consortium directors, 10 programme managers, and four key AAS stakeholders between February and August 2018. These individuals were identified as having first-hand knowledge of the consortia’s establishment and management experiences, and as being best placed to provide insights on the most critical management issues that had arisen over the period. All the interviews were conducted in English, and each lasted for about an hour. Although Skype was the main tool for conducting the interviews, when opportunities for face-to-face interviews came up, such as during DELTAS meetings, these were utilised. The lead author (NT) conducted all the interviews as part of the larger PhD project ^15^. The interviews were semi-structured with topic guides tailored for each type of participant (Extended Data Files 3–5) ^16^. The topic guides were informed by the literature review ^6^ which highlighted the knowledge gaps in the consortium management literature and areas of consortium practice to focus on in empirical work. The data collected in these interviews related to consortia history, management structures, management processes used throughout consortia’s lifecycle, reasons for and influences on these processes, and management successes and challenges. It was essential to consider the positionality of the interviewer (NT) who had several years’ experience in managing HRCS programmes and consortia. This status granted an enhanced understanding of consortia activities and participants’ experiences and unique insights into the research topic. However, we acknowledge that this background could have been a source of biases and assumptions from prior experiences and provided some personal and professional lenses with which the data were considered ^17^. To limit such biases, interview summaries and preliminary interpretations were regularly discussed by the team to ensure that interview questions and presentation of the data were value-neutral and to promote continuous reflexivity.

All the interviews were audio-recorded and transcribed into MS Word (Version 1702) documents, ensuring that all consortium and participant identifiers were replaced with descriptor codes. We used the thematic content analysis approach as it is appropriate for exploratory work and useful for identifying salient issues and recurrent themes from respondents’ accounts ^18^. We employed the ‘directed approach’ to content analysis, which involved starting the process with a few initial coding categories based on the consortium management lifecycle phases and the research questions before inductively identifying emerging codes from the interview data ^19^. The consortium management life cycle phases included pre-inception (period prior to the establishment of consortia), inception (formal establishment), planning (determining processes and activities), and implementation (executing consortia plans) ^20– 22^. Coding was led by the lead author (NT) and reviewed by the other authors at critical points. The coded data were grouped into broad categories and presented by consortia and type of participant in an MS Excel (Version 1702) spreadsheet. We then identified the main themes from the data by identifying patterns, similarities, and differences across consortia and participants. Data from the document review supplemented the data from the interviews during this process.

## Results

We describe the managerial considerations and processes undertaken by participating consortia during the pre-inception, inception, and planning and implementation phases. We also discuss the management expertise and support which consortia leaders draw upon in their management processes.

### Description of consortium management phases and processes

*Pre-inception phase*

The pre-inception phase was an important part of the consortium lifecycle as factors that influenced the formation of consortia emerged during this phase. Except where consortia already existed, the DELTAS Africa funding opportunity triggered formation discussions. In most cases, an established health researcher initiated the discussions on forming a consortium and applying for the DELTAS funding. Several factors motivated the decisions to use the consortium approach. Leaders noted that many African countries faced similar challenges such as infectious diseases (for example, malaria) and inadequate research capacity, and consortia provided the opportunity to synergize and create platforms for pooling resources and consolidating efforts to effectively tackle these common health burdens.

“When you are dealing with a high priority issue that is affecting many countries or many locations, it makes sense to form a consortium… particularly in our environment in low and middle-income countries, because it is clear that none of the countries or the institutions has enough resources.” (Director 5)

The opportunity to capitalise on the diverse strengths of the partners and share research and capacity strengthening experiences and learning was also perceived as an advantage. Although the consortium model was not a pre-requisite for DELTAS programmes, it was encouraged in the call for funding applications, and one of the initiative’s strategic areas focused on fostering networking and collaborations with different stakeholders. Some leaders perceived that applying for the DELTAS funding as a consortium would increase their competitiveness, as collaborations appeared to be favourably considered by funders. Positive experiences and benefits from previous consortia influenced the decision of some leaders to take that route. Many leaders were also committed to strengthening regional and continent-wide (South-South) collaborations and breaking down geographical and language divides in tackling the continent’s health challenges.

“We have very strong institutions in each country and really, we want to work together, having the same scientific objective… we have the same epidemiology in many of these countries. We also want to work in the trans-borders [across countries]… collaborate more with our anglophone countries… There is a real need to put all our strengths together, working together, sharing experiences”. (Director 8)

The leader of the consortium, the director, was usually determined during the pre-inception phase. Typically, the researchers who initiated consortia discussions became the directors. In two of the consortia, the initiative was taken by a group who then nominated leaders based on their individual and institutional capacities. The directors then considered potential partners during the pre-inception phase. Partners recruited at this stage worked with the director to steer the funding application processes. However, the partner selection process was finalised during the consortium inception phase and is discussed in more detail in the next section.

*Inception phase*

Upon award of the funding, the directors mapped out the nature, size (in reference to the number of partners), and structure of the consortia, and completed the partner selection process. Although these steps are discussed in sequence, leaders made multiple decisions concurrently in practice, and different elements influenced each other. For example, geographical factors influenced decisions on partners selected and vice versa.

Regarding the nature of consortia, leaders admitted that they did not deliberate on the type of collaboration being established or its implications for partner expectations and management processes. For many, the term ‘consortium’ was adopted because the funder or previous programmes had used that terminology, and it did not connote any distinctive characteristics beyond representing a group of people working together. Similarly, with the exception of one consortium, leaders did not pre-determine the number of partners; it appeared to be organically derived. Even in the single case where the number of partners was pre-determined, leaders were open to additions if deemed strategically beneficial. Consortium sizes were influenced by a number of factors. Consortia borne out of existing networks often included all interested existing partners and added new partners where additional expertise or demographics were desired. In some cases, expertise and geographical spread were more important in determining partners than a specific size. In other cases, leaders purposed to develop smaller-sized consortia in order to maintain close-knit and manageable collaborations. Funders also influenced consortia size decisions as they recommended fewer partners to avoid unwieldy consortia, and to enhance management and budget efficiency.

“For the programme to cover all twelve members as co-applicants, the budget was too big. So, during the review process, the review committee told us to decrease the budget and focus on a limited number of countries.” (Director 7)

Several factors influenced partner selection. Partners were recruited from existing networks particularly those that leaders had previously worked with on other programmes. Leaders typically reached out to individuals with similar interests and often considered their institution’s scientific and managerial capacities. Some leaders strategically chose institutions with strong research and management capacities to enhance the consortium’s programme performance. Performance in preceding programmes was used as an indicator of capability for partners with past working relationships. Other leaders chose institutions with varying levels of capacity; partners with higher levels of capacity were selected for delivery of programme outputs and mentoring while less-capacitated partners were selected for capacity strengthening opportunities. Geographical and language coverage considerations also informed partner selection decisions. Some leaders restricted the geographical coverage to leverage commonalities such as language and existing regional geo-political linkages. Others expanded the geographical or language reach to leverage epidemiological and other research context diversities and promote inclusivity.

“We wanted to cover much of the diversity of the continent, so we purposely wanted to have a representation of the different parts of the continent that is interested in [Research Area]. We know that the epidemiology is changing; not only is it changing, but it’s different… So, we went and looked for collaborators that can add more diversity to what we are doing.” (Director 7)

It emerged that partner selection decisions were not always straightforward as leaders tried to balance multiple factors which were not always aligned. For instance, when potential partner institutions had disparate scientific and managerial capacities, leaders had to decide which type of capacity to prioritise in their selection.

“We tried to find the strongest PIs and the strongest centres also in terms of management… It doesn’t always match, science and management performance of certain centres, so we have to decide on what we want.” (Manager 10)

Similarly, some leaders wanted to widen the geographical and language reach but were hesitant about working with unknown partners.

“To have a long-standing partnership, we cannot start this with some institution we don’t know; and one of the criteria was that we wanted to breach or fill the gap between geographical regions... and also to break the language barrier.” (Director 10)

The structure of the consortium was also determined during this phase. All consortia had two-tier structures, a decision which was largely influenced by the funder. Consortia had to categorise their partners into two groupings when funders recommended smaller consortia in order to retain all their members. The resulting two-tier consortium structure comprised ‘institutional partners’ or ‘co-applicants’ in the first tier and ‘collaborating partners’ or ‘collaborators’ in the second tier. Co-applicants had part-ownership of the programme, made strategic contributions and significant intellectual inputs, were allocated some of the awarded funds or received sub-awards, and had programmatic responsibilities towards delivering the outputs of the grant ^23^. The collaborators added intellectual and scientific value to the programme and played a minor role in delivering programme outputs ^23^. Collaborators did not typically receive grant funds although their activity costs were usually covered by the lead institution.

“The review committee told us to decrease the budget and to focus on a limited number of countries that are more likely to deliver because the issue of excellence was already part of the criteria. They wanted us to focus on the places which are already capable of delivering.” (Director 7)

“We decided that the group was important, and we put a lot of work into keeping the group together… so, we used those agreed criteria to say, “okay we don’t want anyone to leave… We went with the institutions who met the criteria that the partners themselves had defined. The others stayed on as collaborating partners… And that’s the way that we tried to get two ends to meet. The one end was the expectations expressed by the funders, and the other was our commitment to the consortium as a whole.” (Director 9)

Generally, ‘stronger’ partners were made co-applicants while ‘weaker’ ones became collaborators. Leaders also perceived that having ‘strong’ co-applicants would improve consortia performance as they had the required resources and systems for delivering on the programmatic and budgetary responsibilities.

“These sites were the ones that were considered to be very strong in terms of having strong environments for research and for grant management, and that would help the other sites. So, that was the main consideration.” (Director 4)

“For our first phase… we put all the institutions at the same level, but reporting, administrative issues and the deliverables were very difficult for some of the institutions. So, we have decided this time to take strong institutions as the co-applicants.” (Director 10)

The functions and level of engagement of the two categories were determined by each consortium. So, whereas only co-applicants participated in governing boards in some consortia, this role was open to both categories in others. Collaborators in one consortium were even given programmatic and fiscal responsibilities.

All consortia adopted a hub-and-spoke management model, where the lead institution served as the hub of consortia activities and all other partners were connected to this central point. This model was prescribed by the funder, whose aim was to relate to and hold only the lead institutions accountable for the consortia’s resources and deliverables.

“We deal mostly with the lead institution. Now that was a very deliberate decision we made. So, what we’re promoting is a hub-and-spoke model of consortium management… As the lead institution, you’re the hub. You take charge and responsibility for the resources that we give you on behalf of the entire consortium… we are not directly managing the sub-grantees who are their partners.” (Funder Representative 3)

**Figure 1.**
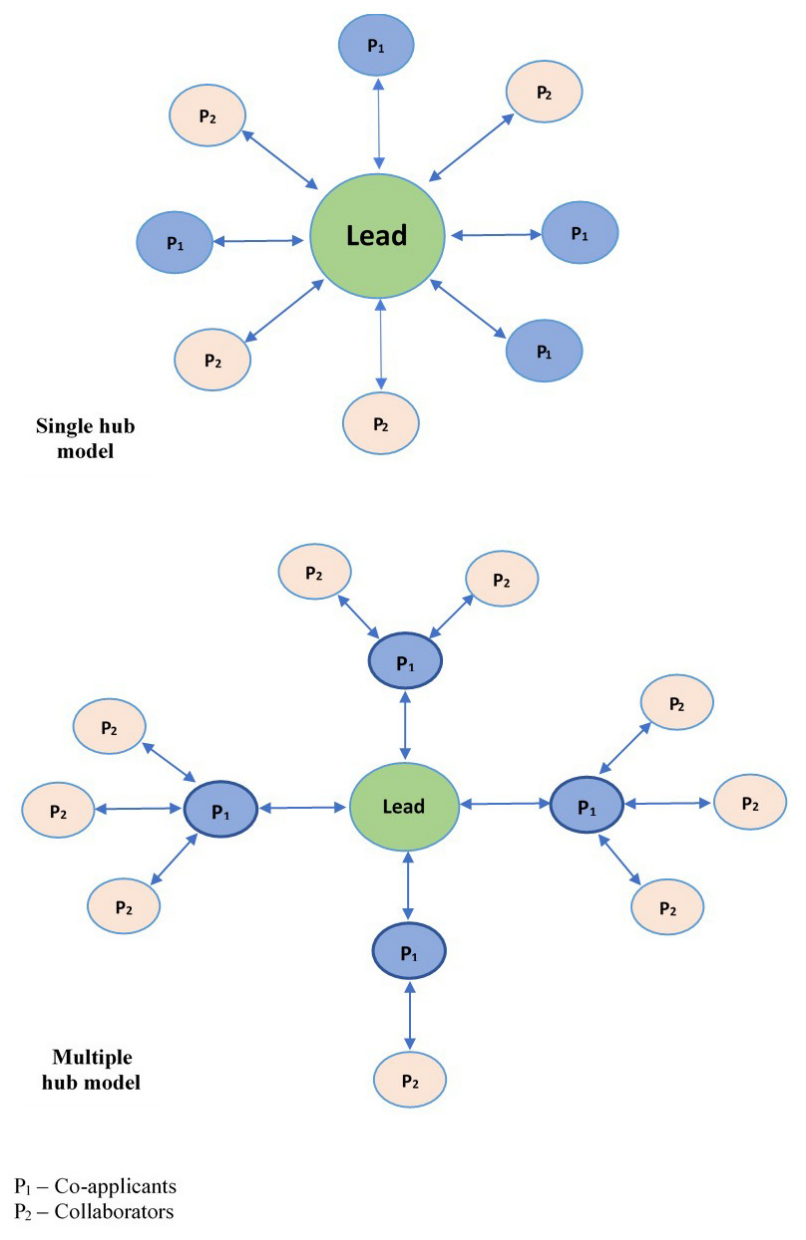
Hub-and-spoke models adopted by consortia.

There were two variants of this model due to the two-tiered structures: the single and multiple hub models ( Figure 1). In the single-hub model, all partners received resources from and reported to the lead institution irrespective of their tier. In the multiple-hub model, co-applicants served as second-level hubs and hence received resources from and reported to the lead institution on behalf of their assigned collaborating institutions. The single-hub model was the most commonly used structure, and only one consortium employed the multiple-hub model. 

*Planning and implementation phase*

Most of the consortium management processes fall in the planning and implementation phase. These include developing goals and activities, assigning roles and responsibilities, instituting governance and management structures, allocating resources, establishing partner management structures, and coordinating and monitoring. We discuss these in turn.

*Developing goals and activities*

Consortia goals and activities were based on the DELTAS Africa strategic areas and partner priorities. Processes for developing consortia goals were mostly participatory, either through a bottom-up or top-down approach. In the bottom-up approach, partners proposed their goals and activities based on their needs, out of which consortium goals were formed in line with the strategic areas. In the top-down approach, consortia leaders developed preliminary consortium goals and activities, which were then proposed to partners for wider discussions and partner inputs.

*Assigning roles and responsibilities*

Roles were primarily determined by partners’ individual and institutional strengths. For example, a partner institution with the human and infrastructural capacity to host and lead training sessions was assigned that role. Naturally, partners with higher capacity levels who were usually co-applicants got bigger roles and responsibilities and ended up getting more resources than collaborators.

“They [co-applicants] get a bigger budget compared to collaborators, and that is linked to their involvement. For example, [Co-applicant X] was hosting a training activity… we supported them to build… So, partners [co-applicants], as I’ve indicated, receive a bigger share because they also give back at a higher level.” (Manager 9)

*Instituting governance and management structures*

**Figure 2.**
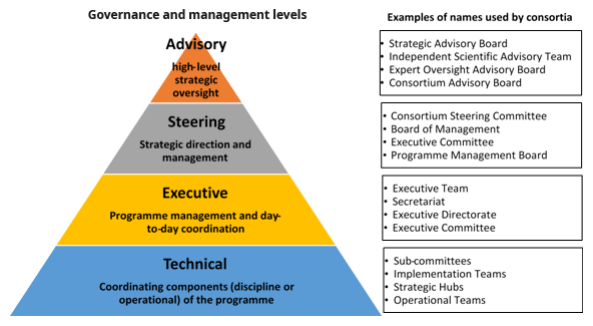
Governance and management structures and designations across the consortia.

Although the consortia individually determined their governance and management structures, they adopted similar structures with only slight naming variations. Four main governance and management levels were used: advisory, steering, executive, and technical ( Figure 2). Advisory level bodies provided high-level strategic oversight and were made up of individuals with the requisite expertise and a wealth of experience who were not members of the participating institutions. The steering bodies were generally made up of partner representatives and participated in management activities including developing policies and processes, allocating resources and monitoring programme progress. The executive teams were responsible for the day-to-day management of consortia activities, operated from a Secretariat which was based in the lead institution. Executive teams included consortium directors, programme managers, and other support staff such as administrative, finance, communication, and monitoring and evaluation (M&E) personnel. The technical groups were responsible for coordinating components of the programme, such as training and M&E. With the exception of a funder requirement to have external and independent advisory boards, consortia chose these governance and management structures themselves. Many leaders noted that these structures were adopted to ensure inclusive and transparent decision-making, and facilitate coordination, monitoring and accountability.

*Allocating resources*

Consortia budgets were developed at the funding application stage and mostly maintained after the grants were awarded. Because of the training focus of the DELTAS programmes, a large percentage of consortia funds were allocated to training fellowships. What differed among consortia was the mode of distribution of the fellowships among partners. Many consortia followed a merit-based system where awards were centrally made based on open competition. A few consortia used a quota system where each partner was given a pre-determined number of fellowships based on equal distribution or partners’ capacity. While the former was centrally managed and the latter was managed at the partner level, competitive selection processes were used for both systems. The difference was the level of competition each system created, with the merit-based system being more competitive. Consortia directors admitted that the decision on which approach to adopt was challenging as each option had its pros and cons. Even when consortia used a similar approach, this was operationalised differently among consortia. For example, some consortia who used the merit-based system devised ways to balance out the award distribution among partners to some extent. Beyond the fellowships, additional funds were allocated to partners based on their institution-specific activities.

“What we do is we decide as a group, i.e., the partners together, on which functions will be managed centrally, and so we put money into that central pool… we know we are going to be training PhDs and postdocs, but the selection is going to be a competitive process… The core budget does not belong to the lead or any partner institution; it’s in the central pool. Then we have allocations that are institutional in nature. In the beginning, we decide on how much will go to this institution and the other institution depending on their needs to some extent, but also the kind of plans that they have.” (Director 5)

*Establishing partner management structures*

Partners were managed at three levels: strategic, operational, and technical. At the strategic level, each partner was represented on the steering committee, ensuring multi-directional accountability among both lead and partner institutions through a ‘peer-management’ system. Partner plans and implementation progress were discussed during these steering committee meetings. At the operational level, the focus was on programmatic and financial management of partners which involved monitoring of consortia activities and partner expenditure and reporting on allocated funds respectively. Consortia either took a primarily centralised approach to partner management or a decentralised approach. For the primarily centralised approach, partner activities and financial transactions were largely coordinated by the Secretariat. Consortia adopting this approach noted that lack of grant management capacity and bureaucracies in partner institutions tended to slow down consortium operations. For the primarily decentralised approach, partners received annual sub-awards based on their work plans and budgets and quarterly disbursements of funds and submitted periodic reports to the Secretariat. Consortia adopting this approach pointed out that it facilitated partner-level activities and involved partners in the management of the grant which further strengthened their capacity.

“The money comes to us, and then we have to disburse the money to our various partner institutions. So, to do that, we need to go into sub-contract with these partner institutions. They have to sign the contract, and then after everything is agreed upon, we send the funds.” (Director 6)

Once again, consortia needed to work through these different options and determine bases for their partner management strategy decisions. It was also observed that some consortia using decentralised systems had some minor managerial elements that were centralised and *vice versa*. However, the dominant approach used by each consortium was always clearly identified and characterised most of their operations. Finally, at the technical level, various committees were formed with representatives from each partner institution to coordinate specific portions of consortia’s scientific or managerial activities. For example, a finance committee was made up of finance personnel from all partner institutions. The ‘peer-management’ system was thus replicated at this level.

*Coordinating and monitoring activities*

The executive teams based at the Secretariats coordinated and monitored consortium-level activities and liaised with partner-level leaders for activities at their respective institutions. Partners submitted annual reports which were consolidated into a consortium report for onward submission to the funder. The Secretariat coordinated these through e-mails, telephone, and online and face-to-face meetings. Consortia also organised annual general meetings (AGM) which provided a platform for leaders, partners, and trainees to meet and offer feedback on both scientific and managerial activities. Advisory and steering committee meetings were also held periodically. In addition, the executive team periodically visited partner sites for monitoring, learning and partner engagement purposes. Overall, progress on consortia activities and outputs were assessed using the DELTAS theory of change indicators, which was considered the ‘standard’ in evaluating consortia performance.

**Figure 3.**
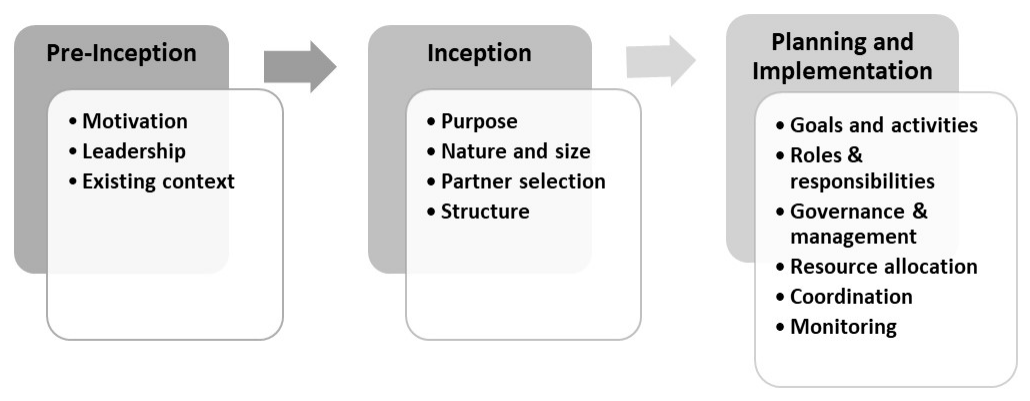
Consortium management phases and processes.

A summary of the management processes described across the three phases are presented in Figure 3. It is important to note that the demarcation between the consortium phases was often blurred. For example, consortia undertook high-level planning for management elements such as partners and their roles, goals and activities, management structures and budgets during the inception phase to inform funding applications. Once funding was obtained and programmes moved into the planning and implementation phase, these provisional decisions were reconsidered. During this phase, greater certainty regarding partners and programme resources and greater clarity on funder expectations facilitated more detailed planning.

### Management expertise and support

None of the consortia directors had received formal training in consortium management. One director noted that they had used a published partnership guide in establishing and managing the consortium and had received some training in accounting and management due to their management position at the host institution. All the directors reported that their expertise was built up over time through participation in other consortia and ‘learning on the job’. Thus, the structures and processes adopted were informed by the previous experiences of the directors and partners and adapted as implementation progressed.

“I didn’t take any classes… I just learnt the hard way. If you miss, the next time you make sure you don’t miss.” (Director 7)

The directors also pointed out that the role of programme managers and other staff of the Secretariat (many of whom had received management-related training), as well as support from members of the wider DELTAS network, and other staff at host institutions were extremely useful. In addition, both directors and managers highlighted the value of training provided by the funding agency including in financial management. Further, some consortia engaged consultants for specific functions (such as M&E) where necessary. Many directors pointed out the importance of strengthening the consortium management skills of consortia stakeholders particularly directors (who are often researchers) using formalized training and resources.

## Discussion

This article seeks to describe the management structures and processes adopted by 10 HRCS consortia of the DELTAS Africa initiative, their approaches to these processes, and some of the factors influencing these practices. We observed that consortia adopted similar management structures and processes. While some of these structures and processes were influenced by the funder, several were determined by the consortia themselves and the similarities appeared to be unplanned. The structures and processes used were also similar to those used by other HRCS consortia published in the literature such as the governance levels, partner selection processes and criteria, and monitoring systems ^6^. Differences were however observed in how consortia approached each management process. For example, some consortia used a top-down approach in determining goals and activities, while others used a bottom-up approach. Similarly, consortia had two categories of partners, co-applicants and collaborators, but the levels of participation accorded the latter varied among consortia. In some consortia, the roles and access to resources given to the two categories were clearly differentiated; in others, there was minimal distinction between the two. Additionally, some consortia used merit-based approaches in allocating resources and others used quota-based approaches; some used a centralised partner management system and others used a decentralised system.

Exploring the management practices of the consortia studied highlighted some key factors that influenced the choice of processes and approaches. Previous experiences played a significant role in management decisions. Consortia mostly opted for ‘the known’ such as preference for previous partners (new partners were often recommended by known partners) and management processes used in previous consortia. In addition, funders had significant influence on consortium management decisions, as their preferences, advanced through recommendations and instructions, were often taken up by consortia. This was demonstrated in the choice of smaller consortia sizes, two-tier structures and partner categorisation, and the use of independent advisory boards. Across the health research and capacity strengthening field, funders have significant, and sometimes even controlling influence on the direction of the programmes they fund ^24– 26^. In the DELTAS consortia, the funder’s emphasis on excellence and preference for supporting stronger institutions shaped many consortia decisions. Thus, institutions with higher levels of research and programme management capacity were prioritised in the selection of partners, almost making existing capacity a pre-requisite for participation in the initiative. Additionally, because high-performing institutions had the capability to take up greater responsibilities, they received more capacity strengthening opportunities such as the number of fellowships, while less-performing institutions received less. It is important to interrogate how this practice aligns with the stated capacity strengthening aims of research capacity strengthening (RCS) programmes. Indeed, some stakeholders are beginning to question the effect of prioritising high-performing institutions when making funding decisions on capacity strengthening and global health equity aims ^27, 28^.

Management decisions were also influenced by a pursuit of research outputs and scientific factors in some cases, such as epidemiological diversity in research studies. There appears to be substantial overlap between conducting research and strengthening research capacity and this is illustrated in how they are currently perceived and evaluated ^29, 30^. Nevertheless, it is essential to differentiate the two, particularly in HRCS consortia, to ensure that management decisions are more capacity-strengthening-oriented than research-oriented. Generally, HRCS programmes are either embedded in broader research programmes (where producing research outputs is the primary aim) or standalone (where capacity strengthening is the primary aim) ^31, 32^. The significance of the different aims on programme decisions and practice as well as programme outcomes have not been adequately explored in the literature ^6^. This study has shown that motivations that drive consortia formation and activities influence the management structures and processes that are adopted. Hence, it would be essential to assess in more depth the linkages between consortia’s motivation for formation, their primary aims, management practices, and consortia outcomes.

It has become clear that consortia often face dilemmas as they make management decisions, as demonstrated during partner selection, resource allocation, and partner management. Decision-making was not always straightforward as leaders tried to balance divergent factors. The existence of dilemmas in decision-making appears to be a common management phenomenon across many fields ^33, 34^. Several strategies for managing dilemmas have been proposed in the broader management literature such as adopting and consistently applying a guiding philosophy ^35^ and transcending the current options by developing a more advanced range of understandings and behaviours ^34, 36^. It will be instructive to generate some RCS-specific guidance than can assist consortia in resolving emerging dilemmas.

From this study, it was evident that consortia’s approaches to management were unique due to their varying contexts and influences. For example, no two consortia adopting the merit-based resource allocation system operationalised it the same way. Also, consortia using decentralised partner management systems incorporated certain centralised elements in their processes albeit minimally, and *vice versa*. Thus, it would be too complex to place consortia in clear-cut categories, as any such categorization may miss the intricate distinctions across consortia and fail to depict the true picture of each consortium’s management approach. This highlights the unique, contextualized, and nuanced nature of management approaches, and demonstrates that consortium management approaches cannot easily be formularized or overly prescriptive. However, it would be important to draw out key considerations and develop evidence-based frameworks that can guide HRCS consortia in their decision-making processes.

Finally, this study has highlighted the importance of flexibility in consortium management. Many of the adopted management structures, processes and practices were continuously refined as consortia activities evolved. Although consortia proposed initial management plans, these were adapted in latter stages as inputs were made by funders during review and reporting processes, and by partners when they got involved in the steering committees. Furthermore, management processes and practices continued to be refined, informed by feedback from the implementation stage. Considering the evolving nature of consortium management, it would be valuable to embed learning frameworks within consortia M&E structures to enhance evidence-informed evolution of management practice. In recent times, emphasis has been made on learning in M&E frameworks (which are now commonly referred to as monitoring, evaluation and learning frameworks). The aim of the ‘learning’ component is to deliberately and continuously improve the processes and outcomes of an intervention to ensure that they are relevant, efficient, and effective ^37, 38^. Incorporating management-specific learning will be particularly valuable for strengthening consortia considering the lack of formal management training of most leaders. Such learning, when documented, will also form the basis for evidence-based guidance for consortium management.

This study was not without limitations. We acknowledge that the structures, processes, and practices described in this paper pertain to consortia in one initiative (DELTAS Africa) and could have been significantly influenced by the initiative’s design and the funders stipulations on how consortia should be managed. However, the similarities between the management structures and processes used by the DELTAS consortia and those identified in the literature indicate that these practices are common among HRCS consortia. Nonetheless, it would be beneficial to conduct similar studies with consortia in different initiatives and geographical contexts such as consortia led by high-income countries where the capacity disparities between the leaders and other partners might be greater.

## Conclusion

Consortia are widely used in implementing HRCS initiatives and this study aimed to fill a gap in the published literature regarding how HRCS consortia are managed. This exploratory study has identified the management processes used by HRCS consortia and drawn attention to what informs these processes as well as the challenges leaders face in managing consortia. The study has pointed out the need to empirically answer consortium management questions such as: 1) how to determine consortia characteristics and the implications of different options; 2) what factors should have greater influence when making management decisions (such as when selecting partners); and 3) which management approaches work better (for example centralised or decentralised, merit-based or quota based) and in what contexts. The findings have highlighted the need for more in-depth work on the different management approaches, factors that influence the choice of approaches, and the implications of each approach on consortia’s goals. The study has also underscored the need to distinguish between research aims and research capacity strengthening (RCS) aims and their influences on management decisions. Finally, the findings have indicated that it is important to empirically ascertain how the management practices of HRCS consortia influence their capacity outcomes as this will strengthen the evidence on consortia practice and inform the design and management of HRCS programmes.

## Data availability 

### Underlying data

Access to underlying data is restricted to the study team for ethical reasons. Due to the unique characteristics of participating consortia and the small number and distinct roles of study participants, anonymization of study data does not adequately ensure the privacy and protection of study participants. Hence, the request for permission from the African Academy of Sciences to engage the DELTAS consortia and the application for ethical approval included an explicit statement on restriction of the raw data to the study team. Specific requests can be submitted to the KWTRP Data Governance Committee via email: dgc@kemri-wellcome.org.

### Extended data

Harvard Daraverse. DELTAS Consortia Processes Supplementary Files. https://doi.org/10.7910/DVN/IUBWDI ^16^.

This project contains the following extended data:

- Extended Data File 1 - DELTAS Consortia Processes Document Review Checklist
- Extended Data File 2 - DELTAS Consortia Processes Template for Consortia Data
- Extended Data File 3 - DELTAS Consortia Processes KII Topic Guide for Directors
- Extended Data File 4 - DELTAS Consortia Processes KII Topic Guide for Managers
- Extended Data File 5 - DELTAS Consortia Processes KII Topic Guide for Funding Actors

Data are available under the terms of the Creative Commons Zero "No rights reserved" data waiver (CC0 1.0 Public domain dedication).
