# Supplementary material for: Consortium management structures, processes, and approaches: The DELTAS Africa example
Source: Wellcome Open Res. 2022 Apr 21;7:139. [Version 1] doi: 10.12688/wellcomeopenres.17721.1 (PMC10474346; doi:10.12688/wellcomeopenres.17721.1)
Supplement: Peer review history

# Reviewer report: Reviewer response for version 1

**DOI:** 10.21956/wellcomeopenres.19609.r65166

**Published:** 2023-09-25

**Authors:** Ronald Kiguba

**Affiliations:** 1 Makerere University, Kampala, Uganda

Thank you for giving me the opportunity to review the manuscript titled ‘Consortium management structures, processes, and approaches: The DELTAS Africa example’. The manuscript is very well written and benefits from the extensive experience of the lead author having worked in a similar environment for a relatively long time. The approach to data collection and analysis is comprehensive and I have nothing much to add.

 Congratulations to the authors on the job well done!

Is the work clearly and accurately presented and does it cite the current literature?

Yes

If applicable, is the statistical analysis and its interpretation appropriate?

Not applicable

Are all the source data underlying the results available to ensure full reproducibility?

Yes

Is the study design appropriate and is the work technically sound?

Yes

Are the conclusions drawn adequately supported by the results?

Yes

Are sufficient details of methods and analysis provided to allow replication by others?

Yes

Reviewer Expertise:

NA

I confirm that I have read this submission and believe that I have an appropriate level of expertise to confirm that it is of an acceptable scientific standard.

# Reviewer report: Reviewer response for version 1

**DOI:** 10.21956/wellcomeopenres.19609.r65177

**Published:** 2023-09-01

**Authors:** Mona Duggal, Gurjinder Kaur Brar

**Affiliations:** 1 Advanced Eye Centre, Post Graduate Institute of Medical Education and Research, Chandigarh, India; 2 Haematology, Post Graduate institute of Medical Education and Research, Chandigarh, Chandigarh, India; 3 Department of Hematology, Post Graduate Institute of Medical education and Research, Chandigarh, Chandigarh, India

The study conducted by Tagoe *et al.*, is one of a kind to identify the consortium management processes employed by 10 DELTAS Africa consortia, factors influencing programme outputs and outcomes, and leaders’ consortium management experiences. The authors very well used the consortium management life cycle phases which included pre-inception (period prior to the establishment of consortia), inception (formal establishment), planning (determining processes and activities), and implementation (executing consortia plans).

 The authors can give more details of the methods and analysis to allow replication by others. A detail about different themes and sub-themes can be given in the methodology which can be further used in the results. 

 Again I want to congratulate all the authors for putting their handwork for conducting this study.

Is the work clearly and accurately presented and does it cite the current literature?

Yes

If applicable, is the statistical analysis and its interpretation appropriate?

Not applicable

Are all the source data underlying the results available to ensure full reproducibility?

Yes

Is the study design appropriate and is the work technically sound?

Yes

Are the conclusions drawn adequately supported by the results?

Yes

Are sufficient details of methods and analysis provided to allow replication by others?

Partly

Reviewer Expertise:

I have a wide experience of implementation research. My area of expertise is Public health,Nutrition, Fortification, Community ophthalmology and maternal child health etc.

We confirm that we have read this submission and believe that we have an appropriate level of expertise to confirm that it is of an acceptable scientific standard.

# Reviewer report: Reviewer response for version 1

**DOI:** 10.21956/wellcomeopenres.19609.r54216

**Published:** 2023-02-16

**Authors:** Sara Jewett

**Affiliations:** 1 Division of Health & Society, School of Public Health, University of the Witwatersrand, Johannesburg, South Africa

The manuscript entitled ‘Consortium management structures, processes, and approaches: The DELTAS Africa example’ provides a well-structured analysis of how 10 African-led consortia approached questions of management at three key stages: pre-inception, inception, and planning and implementing. As part of a PhD, the researchers applied a qualitative exploratory study design that involved 24 key informant interviews with consortia directors, managers and funders, supplemented by a document review.

 My assessment is that the research is clearly and accurately presented, citing current literature. The study focus is justified, with increasing funder interest in locally-led health research capacity strengthening (HRCS) in low and middle income country (LMIC) contexts. While consortia have been reporting and publishing on key outputs and outcomes, such as numbers of students trained and research published, differences in management structures and processes that may influence consortia goals has not received the same attention in the peer reviewed literature. The authors draw on relevant and recent literature to locate their study within a larger discourse.  

 In terms of results and discussion, the authors provide constructive insight into the thinking of leaders at the helm of HRCS, at least in the context of the DELTAS initiative. A key strength of this article is that the authors go beyond simple observations of different management models, such as two variations of the hub and spoke management model, to delve deeper into questions of power and influence.

 The key take away messages for me are how funding priorities and past experiences influence the selection of consortium members early in the process. The study authors highlight how pragmatism, sticking with what one knows, can operate against bringing in relatively newer or weaker capacity institutions. They also discuss the tension between consortia centered on capacity-strengthening vs research and how this influences choices on who gets invited to participate. The variations in how different consortia allocated decision-making power and resources is particularly compelling, illustrating how pragmatism and values can be at odds. The study findings also underline the importance of context. The authors adeptly link examples from DELTAS back to broader discussions of how funder and recipient priorities contribute to (or undermine) the aims of global health equity. The study also contains practical insights, such as finding that most consortia directors have not received any formal training on managing consortia. This type of information presents obvious opportunities for follow-up consultations about whether such training is desired and needed.

 The manuscript itself is well organized and has a logical flow. I appreciate that the first author is herself an African researcher and I applaud her work. I have a few suggestions for strengthening specific sections. These are relatively minor recommendations, which I believe may strengthen an already strong contribution to literature on this topic.

 A few points to consider for the introduction:

1. Evaluation was not explicitly explored as a stage or depicted as being part of management processes (Figure 3). Despite this omission in the framing of the article, the authors address the importance of evaluation and/or learning in the discussion. Consider whether you want to mention evaluation earlier.
2. Fix the website link provided in the study setting section. It currently gives an error message.
3. The DELTAS theory of change is mentioned in a few places. Please provide a citation or link where this can be found.

The study design is suitable for this type of study. While the authors provide many details on the methods and analysis, there are a few ways this part of the manuscript can be improved in terms of replication and rigor.

1. Use active vs passive voice in the section on data collection and analysis. The current use of passive voice makes it difficult to know if one or more people was involved at any given point, particularly with the data extraction.
2. The presentation of the first author's positionality is great. Given that the co-authors were involved in analysis to ‘ensure that interview questions and presentation of the data were value-neutral’, their positionality should also be reported. As an aside, I think a demonstration of being value-aware vs.value-neutral is more realistic to claim in this kind of analysis, but it is a point for the authors to argue in the context of their positionality.

Generally speaking, the results are well structured and substantiated through relevant quotes. There is one quote segment that is either repeated twice on  p.5 or the same person used the same words twice: ‘The review committee told us to decrease the budget and to focus on a limited number of countries.’ In either case, one instance should be removed or replaced. Otherwise, I enjoyed this section, including the visuals.

 The fact that the findings are constrained to a single initiative is noted as a limitation. Qualitative designs are not meant to be generalizable and the authors have identified where they think lessons could be transferable by citing literature, which is appropriate. There is no action needed. I just want to affirm this position.

 Finally, the authors have argued that they cannot make the full transcripts or extracted data publicly available due to concerns about anonymity. I am convinced by their arguments and do not have a problem with this point, as they have included the study tools in supplemental files and verbatim quotes in text to substantiate the results.

Is the work clearly and accurately presented and does it cite the current literature?

Yes

If applicable, is the statistical analysis and its interpretation appropriate?

Not applicable

Are all the source data underlying the results available to ensure full reproducibility?

No

Is the study design appropriate and is the work technically sound?

Yes

Are the conclusions drawn adequately supported by the results?

Yes

Are sufficient details of methods and analysis provided to allow replication by others?

Partly

Reviewer Expertise:

My personal area of research is infant feeding and commercial determinants of health, but I have also participated in and published about health research capacity strengthening in Africa.

I confirm that I have read this submission and believe that I have an appropriate level of expertise to confirm that it is of an acceptable scientific standard.
